# Roughness and Gloss of 3D-Printed Crowns Following Polishing or Varnish Application

**DOI:** 10.3390/ma18143308

**Published:** 2025-07-14

**Authors:** Silvia Rojas-Rueda, Tariq Aziz Alsahafi, Mohammed Hammamy, Neeraj Surathu, Nitish Surathu, Nathaniel C. Lawson, Taiseer A. Sulaiman

**Affiliations:** 1 Department of Clinical and Community Sciences, School of Dentistry, University of Alabama at Birmingham, Birmingham, AL 35209, USA; srojasru@uab.edu (S.R.-R.); mhammamy@uab.edu (M.H.); 2 Department of Biomedical Sciences, Adams School of Dentistry, University of North Carolina at Chapel Hill, Chapel Hill, NC 27599, USA; alsahafi@email.unc.edu; 3 Department of Conservative Dentistry, College of Dentistry, Qassim University, Qassim 52571, Saudi Arabia; 4 The ACE Institute, Hamilton 3210, New Zealand; neeraj.surathu24@gmail.com (N.S.); drnitish@gmail.com (N.S.); 5 Department of Restorative Sciences, Adams School of Dentistry, University of North Carolina at Chapel Hill, Chapel Hill, NC 27599, USA; sulaiman@unc.edu

**Keywords:** dental crowns, 3D printing, surface roughness, polishing, gloss

## Abstract

The aim of this study was to evaluate and compare the surface roughness and gloss—both initially and after simulated toothbrushing—of three 3D-printed crown materials subjected to different surface treatments: varnishing, polishing with diamond-impregnated rubber polishers, and polishing with a bristle brush and paste. Disc-shaped specimens (n = 90) were 3D-printed using three commercially available crown resins (Rodin Sculpture, VarseoSmile TriniQ, and OnX Tough 2) and post-processed per manufacturers’ instructions. Specimens were divided into three surface treatment groups: application of a light-cured varnish, polishing with a two-step diamond-impregnated rubber polisher, or polishing with a bristle brush and abrasive paste. Surface roughness and gloss were measured after treatment and again following 20,000 cycles of simulated toothbrushing. Additional specimens were prepared for Vickers microhardness testing and determination of filler weight percentage (wt%). Statistical comparisons were performed using two-way ANOVA with significance set at *p* < 0.05. Results: The varnish provided the statistically lowest roughness of all surface treatments for all materials. The bristle brush and abrasive paste polishing protocol produced the greatest gloss for the softest material (VarseoSmile TriniQ) and lowest gloss for the hardest material (Rodin Sculpture), whereas the two-step diamond-impregnated rubber polisher produced an equivalent gloss on all materials. Following toothbrushing, roughness was minimally affected; however, gloss was considerably reduced. Conclusions: All tested polishing and varnishing methods achieved clinically acceptable surface roughness (Ra < 0.2 µm) that persisted after simulated toothbrushing. Notably, the two-step diamond-impregnated rubber polisher produced consistent gloss across all materials, while the bristle brush and abrasive paste polishing protocol performed better on softer materials, and varnish application resulted in equal or superior gloss and roughness retention compared to polishing.

## 1. Introduction

Achieving an esthetically pleasing polish and a smooth surface are key clinical objectives with 3D-printed crown materials. Higher gloss enhances the esthetic appeal of a dental restoration [1]. A smoother surface will reduce plaque accumulation [2], decrease staining [3,4], and improve patient comfort [5]. The desired surface properties can be accomplished through polishing; however, the lower filler content in 3D-printed crowns, compared to other resin composites, necessitates further research to determine the most effective polishing method for 3D-printed materials with varying filler contents.

The filler percentage in 3D-printed crown materials can range from 0% to around 60%. As a result, their hardness is typically between 15% and 50% that of a conventional restorative composite [6]. This lower filler content can influence the choice of the most effective polishing system for these materials. The effectiveness of polishing systems is largely dependent on the hardness of the abrasive particles (such as aluminum oxide or diamond) in relation to the surface being polished [7]. Furthermore, the force applied during polishing can impact its success, with one study reporting that increased polishing force may actually reduce its effectiveness [8]. Consequently, the tool used to apply the abrasive (e.g., bristle brush or rubber point) may affect the force applied, thereby influencing the polishing outcome. Previous studies have demonstrated that both bristle brushes and rag wheel with polishing pastes are effective polishing tools for provisional crowns [9,10]. For highly filled resin composites, polishing is most effectively achieved with aluminum oxide or diamond-impregnated brushes, plastic discs, or rubber points [7,11,12,13]. A previous study reported that a goat hair brush and diamond paste polishing system produced a smoother surface than a silicone polisher on a relatively low-filled 3D-printed material (reported at 30–50% filled) [14,15]. Some 3D-printed materials have now achieved 60% filler content [16] and, therefore, may require a different polishing protocol. Varnishing, which is the application of a low-viscosity resin-based material, is a more efficient workflow for creating a smooth surface than polishing. Previous studies have reported similar roughness achieved with varnishing as could be achieved with polishing [14,15,17].

The durability of 3D-printed crown materials over time depends in part on their capacity to retain surface polish during prolonged clinical use. Resin-based materials are softer than ceramic materials, which makes them prone to toothbrush wear [6,18,19]. A previous study reported that a 50% filled 3D-printed crown material experienced a significantly greater loss in gloss and increase in roughness relative to a milled or light-polymerized composite [19].

No previous studies have compared the effectiveness of different polishing systems on 3D-printed resins of different filler concentrations. The purpose of this study is to compare the roughness, gloss, gloss after toothbrushing, and polish after toothbrushing achieved by three different 3D-printed crown materials following application of a varnish or two different polishing protocols (diamond-impregnated rubber polishers and a bristle brush polisher with an abrasive paste). The novelty of this study lies in evaluating whether different polishing protocols vary in effectiveness depending on the filler content of the 3D-printed crown materials. The null hypotheses are that there will be no differences in any of the tested properties for either the type of material, the type of surface treatment, or their interaction with each other.

## 2. Materials and Methods

### 2.1. Specimen Preparation

Disc-shaped specimens (12 mm diameter × 3 mm thickness) were designed using CAD software (Autodesk Tinkercad, San Francisco, CA, USA, www.tinkercad.com, accessed 1 January 2024) and printed using three 3D-printed resins (Table 1): Rodin Sculpture (Pac-Dent; Brea, CA, USA), OnX Tough 2 (SprintRay; Los Angeles, CA, USA), and VarseoSmile TriniQ (BEGO GmbH & Co; Bremen, Germany). Printing of Rodin Sculpture and VarseoSmile TriniQ was performed using an Ackuretta SOL printer (Ackuretta Technologies, Taipei, Taiwan). Printing of OnX Tough 2 was performed with a Pro 2 printer (SprintRay, Los Angeles, CA, USA). The resins were selected as commonly used materials representative of various filler wt%. The printers were selected based on which printer was validated to print each resin. The circular faces of the discs were oriented parallel to the build plate to mimic the orientation of the crown positioned to avoid supports on its intaglio surface. Specimens were printed with a layer thickness of 100 µm.

Specimens were post-processed based on their manufacturer’s recommendations. Rodin Sculpture specimens were wiped with a paper towel dampened with 99% isopropyl alcohol until a clean, matte finish was achieved. VarseoSmile TriniQ specimens were cleaned in 99% isopropyl alcohol in an ultrasonic machine (Vector 55, Jelenko, San Diego, CA, USA) for a 3 min and then 2 min cycle and dried with compressed air. OnX Tough 2 specimens were hand-sprayed with 91% isopropyl alcohol, wiped with a dry towel for 15 s, blow-dried for 30 s, and the spray and wipe step was repeated. Specimens were polymerized in a curing unit (Curie Plus; Ackuretta for Rodin Sculpture and VarseoSmile TriniQ and NanoCure; SprintRay for OnX Tough 2) based on their manufacturer’s recommendations.

### 2.2. Specimen Surface Treatment

**Figure 1:**
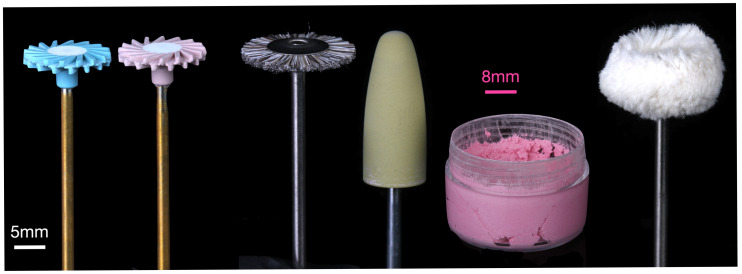
Polishers used in this study (**left** to **right**): ASAP pre-polish, ASAP high-shine, horsehair bristle brush, rubber acrylic polisher, diamond abrasive paste, and cotton buff wheel. Note: pink scale bar for abrasive paste and white scale bar for all others.

Specimens were divided into three groups: application of a light-cured varnish (Rodin Glaze N2-Free, Pac-Dent, Brea, CA, USA), polishing with a 2-step diamond-impregnated rubber polishing kit (A.S.A.P. Indirect+ All Surface Access Polishers; Clinicians Choice, Lompoc, CA, USA), and polishing with a polishing kit (MOD 3D-printed and milled acrylic polishing kit; Komet, Lemgo, Germany) including a bristle brush and abrasive paste (MOD polish; MOD, Charleston, SC, USA) (Figure 1). An a priori power analysis was conducted using G*Power (version 3.1) to determine the required sample size for a two-way ANOVA with two fixed factors (material and surface treatment), each with three levels. Assuming a large effect size (f = 0.40), an alpha level of 0.05, and a desired power of 0.80, the analysis indicated that a total sample size of 66 specimens would be sufficient to detect significant main effects or interactions. The large effect size (f) was chosen using group means and standard deviations reported in a prior study [19] by calculating the ratio of the standard deviation of the group means to the pooled within-group standard deviation (2.1 for roughness and 3.7 for gloss). To ensure sufficient power and account for potential data loss or variability, a sample size of 10 specimens per group (total n = 90) was selected, which exceeds the minimum required sample size from the power analysis and provides a more robust test of main and interaction effects.

Varnished specimens were coated with a thin coat of a light-cured varnish (Rodin Glaze N2-Free) applied using a fine-tipped synthetic brush and polymerized in a curing unit (Curie Plus, Ackuretta, Taipei, Taiwan) following the manufacturer’s instructions.

The first polishing group used a two-step rubber polishing system applied without water, beginning with a blue disc (pre-polish) followed by a pink disc (final high-shine), both operated in a laboratory handpiece at 10,000 RPM. The second group followed a polishing protocol involving a diamond abrasive paste. This procedure began with a horsehair bristle brush (AR9463.HP.190, Komet) at 10,000 RPM in combination with an abrasive paste (MOD Polish Ultra Fine, MOD), followed by a rubber acrylic polisher (9489M.HP.100, Komet) at the same speed and using the same abrasive paste. Both groups completed polishing with a cotton buff wheel (9628.HP.220, Komet) at 10,000 RPM, used without abrasive paste. Each polishing step was performed for 30 s by a single operator using circular motions. The operator periodically calibrated the applied force using a scale to maintain an approximate polishing force of 2 N. No other training was performed for the operator and the operator was not blinded to the polishing protocol. Surface roughness and gloss were measured after each successive polishing step; however, only the values from the final step were used for statistical analysis. Representative specimens were gold-coated and analyzed with a scanning electron microscope (SEM; Quanta FEG 650, FEI, Hillsboro, OR, USA) secondary electron imaging mode. Representative sections of the polishers were also examined with SEM and elemental composition of the abrasive particles was conducted using energy-dispersive spectroscopy (EDS). Representative specimens from the varnished specimens were sectioned and imaged with an optical microscope (VR-6100; Keyence Corp., Tokyo, Japan). The thickness of the varnish was measured with built-in image analysis.

### 2.3. Specimen Measurement

Surface roughness measurements were performed using a contact profilometer (SJ-210; Mitutoyo, Kawasaki, Japan) equipped with a 5 µm stylus tip and a 4 mN measuring force. An initial scan was performed on the specimens in order to determine an approximate roughness value (Arithmetic Average Roughness, Ra). Based on the initial scan, a sample length of 4 mm (Ra > 0.1) or 1.25 mm (Ra < 0.1) and a cut-off filter of 0.8 mm (Ra > 0.1) or 0.25 mm (Ra < 0.1) were utilized. All measurements were performed by a single operator to ensure consistency.

Surface gloss was measured at a 60° incidence angle using a Novo-Curve Glossmeter (Rhopoint Instruments, St Leonards, UK) with a 4.7 × 2 mm measurement window, calibrated before each session. Two readings were taken at 2 different perpendicular rotations per specimen. All measurements were performed by a single operator to ensure consistency.

### 2.4. Toothbrushing

The varnished or polished specimens from each group were secured in removable holders compatible with a toothbrush simulator (ZM 3.12, SD Mechatronik, Feldkirchen-Westerham, Germany). Each specimen was mounted onto a glass slide and positioned to ensure a flat, even surface orientation during testing. A manual toothbrush (Disposable Soft Bristle Toothbrush; Navona, Beijing, China) was attached to the simulator so that the bristles aligned directly over the specimen surface. A constant force of 350 g was applied by the simulator, and a new toothbrush head was used for every specimen. A toothpaste slurry was prepared using Crest ProHealth (Procter & Gamble; Cincinnati, OH, USA) mixed with distilled water at a 2:1 water-to-toothpaste ratio. The slurry was added to each holder to fully cover the specimen surface and was refreshed every 10,000 cycles. Toothbrushing was simulated using a 10 mm linear motion at 2 Hz for 20,000 cycles. Roughness and gloss were re-measured as previously described.

### 2.5. Statistical Analysis

The final roughness, gloss, roughness after toothbrushing, and gloss after toothbrushing were analyzed to assess statistically significant differences among groups. Normality was assessed by using the Shapiro–Wilk normality test, and homogeneity of variances was confirmed by the Levene test. Two-way ANOVAs were performed for each property to identify the effects of material and surface treatment, using SPSS software version 24 (IBM, Armonk, NY, USA). All analyses were performed with a significance level set at 5% (*p* < 0.05).

### 2.6. Characterization of the 3D-Printed Materials

Additional specimens of each 3D-printed material were fabricated following the same procedure as previously outlined for the purpose of evaluating microhardness and filler weight percentage (wt%). Vickers microhardness testing was conducted using a microhardness tester (Model 900, Phase II, Upper Saddle River, NJ, USA). Each disc was wet-polished using silicon carbide paper up to 1200 grit to standardize the surface finish. For each material, three specimens (n = 3) were tested, with five indentations per disc, yielding 15 measurements per group. A Vickers diamond indenter was used with a 0.98 N load applied for 15 s. Hardness values (HV) were calculated using the formula: HV = 0.18544 × P/d^2^, where P is the applied load in Newtons and d is the mean diagonal length in millimeters.

For filler content analysis, the discs were placed in high-alumina crucibles (20 mL, Coors, Sigma Aldrich, St. Louis, MO, USA), and the initial mass (W_0_) was recorded using an analytical balance with 0.0001 g precision (AE163, Mettler Toledo, Columbus, OH, USA). The specimens (n = 5) were heated in a muffle furnace at 800 °C for 30 min to thermally degrade the organic resin matrix. After a 15 min cooling period, the residual inorganic filler was weighed (W_1_) and the filler weight percentage was determined using the equation: Filler wt% = (W_1_/W_0_) × 100%.

## 3. Results

**Figure 2:**
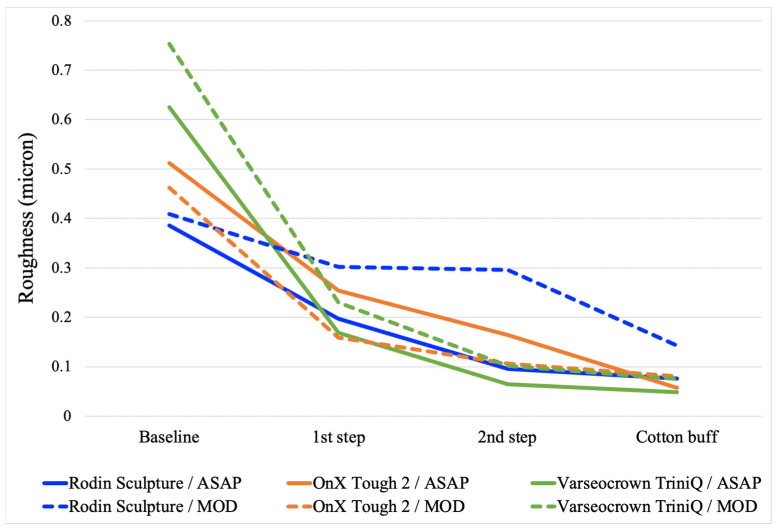
The average roughness for each step of two polishing protocols on three 3D-printed crown materials. For the ASAP system, the 1st step is the blue pre-polisher, and the 2nd step is the pink high-shine polisher. For the MOD system, the 1st step is the bristle brush with polishing paste, and the 2nd step is the rubber acrylic polisher with polishing paste.

**Figure 3:**
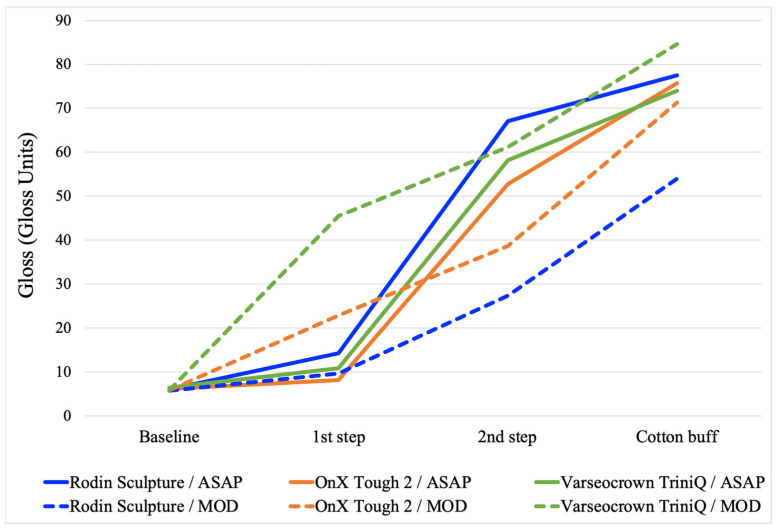
The average gloss for each step of two polishing protocols on three 3D-printed crown materials. For the ASAP system, the 1st step is the blue pre-polisher, and the 2nd step is the pink high-shine polisher. For the MOD system, the 1st step is the bristle brush with polishing paste, and the 2nd step is the rubber acrylic polisher with polishing paste.

The average roughness and gloss for each step of both polishing protocols are presented in Figure 2 and Figure 3. The final roughness, gloss, roughness after toothbrushing, and gloss after toothbrushing for all surface treatments and all materials are presented in Table 2. The two-way ANOVAs determined that material, surface treatment, and their interaction were all significant for each property (Table 3, Table 4, Table 5 and Table 6). For all properties, an analysis of simple main effects for material and surface treatment was performed with statistical significance receiving a Bonferroni adjustment. The results of the pairwise comparisons are presented in Table 2. The microhardness and filler wt% of the materials are listed in Table 1.

**Figure 4:**
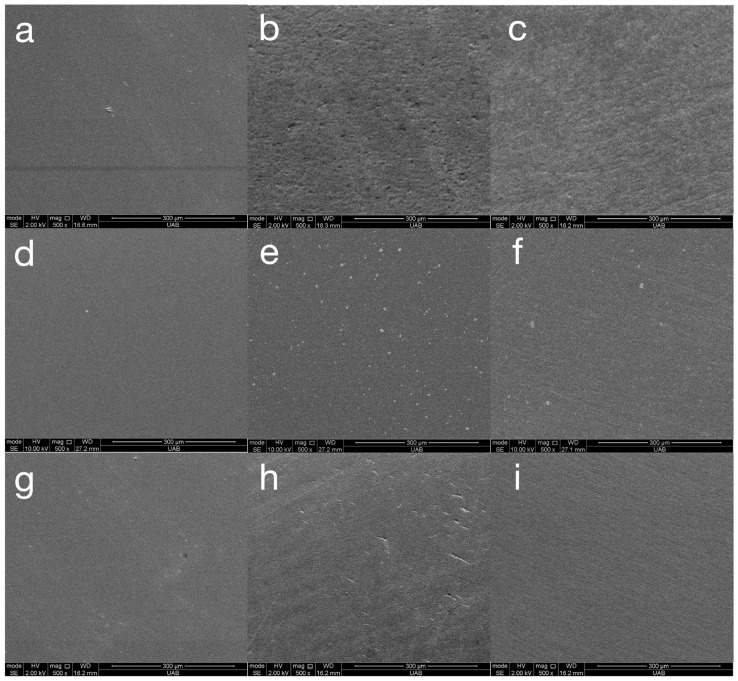
Representative (n = 1) SEM images (original magnification of 500×) of materials Rodin Sculpture (**a**–**c**), OnX Tough 2 (**d**–**f**), and VarseoSmile TriniQ (**g**–**i**) with different surface treatments of varnish (**a**,**d**,**g**), ASAP (**b**,**e**,**h**), and MOD (**c**,**f**,**i**).

**Figure 5:**
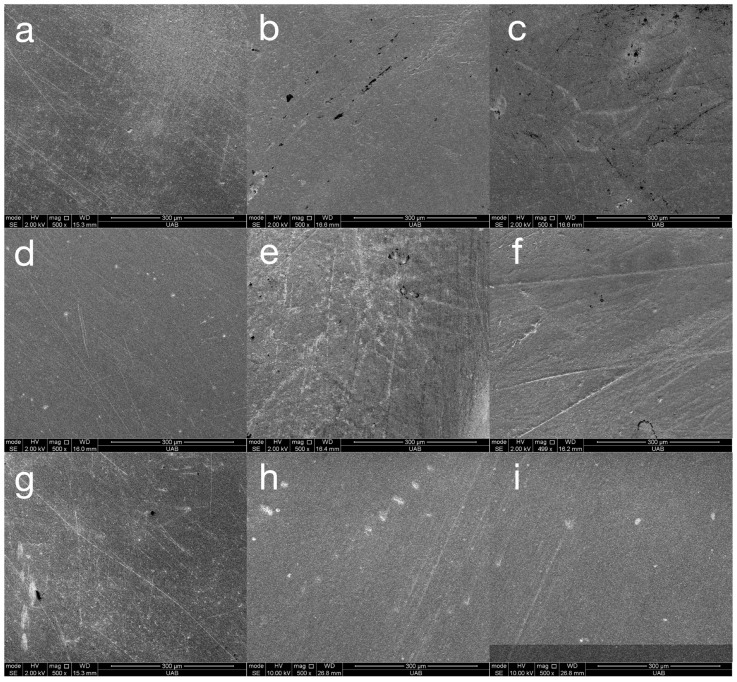
Representative (n = 1) SEM images (original magnification of 500×) of toothbrushed materials Rodin Sculpture (**a**–**c**), OnX Tough 2 (**d**–**f**), and VarseoSmile TriniQ (**g**–**i**) with different surface treatments of varnish (**a**,**d**,**g**), ASAP (**b**,**e**,**h**), and MOD (**c**,**f**,**i**).

**Figure 6:**
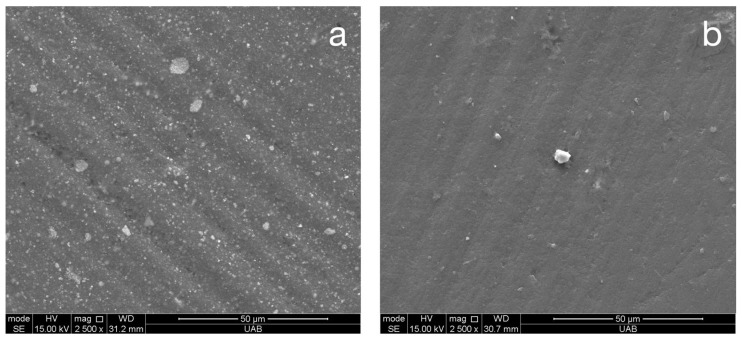
Representative (n = 2) high-magnification SEM images (original magnification of 2500×) of scratches created by toothbrushing from a representative specimen of Rodin Sculpture (**a**) and VarseoSmile TriniQ (**b**).

Representative SEM images of all materials following the final step of each surface treatment are presented in Figure 4. These images are included only to validate the roughness and gloss data. The varnish coating reveals a completely smooth surface for all materials. The ASAP polishing system reveals a smooth but pitted surface for Rodin Sculpture and a pitted surface with widely spaced fine scratches for VarseoSmile TriniQ. The MOD polishing system reveals widely spaced fine scratches for Rodin Sculpture and closely spaced very fine scratches for OnX Tough 2 and VarseoSmile TriniQ. In general, all of the surfaces are relatively smooth, validating the low Ra values measured in this study. The SEM images of materials after toothbrushing are presented in Figure 5. These images demonstrate fine, shallow, irregularly dispersed scratches throughout the specimens. Although there was a general trend of the toothbrushing scratches being oriented in a similar direction, the scratches were not always linear or parallel. Higher magnification images of the scratches from toothbrushing reveal that they are shallow and blunted (Figure 6). They appear to result from indentation ploughing wear rather than traditional scratching wear, likely due to the low modulus of the 3D-printed material, which promotes material displacement over material fracture and removal.

**Figure 7:**
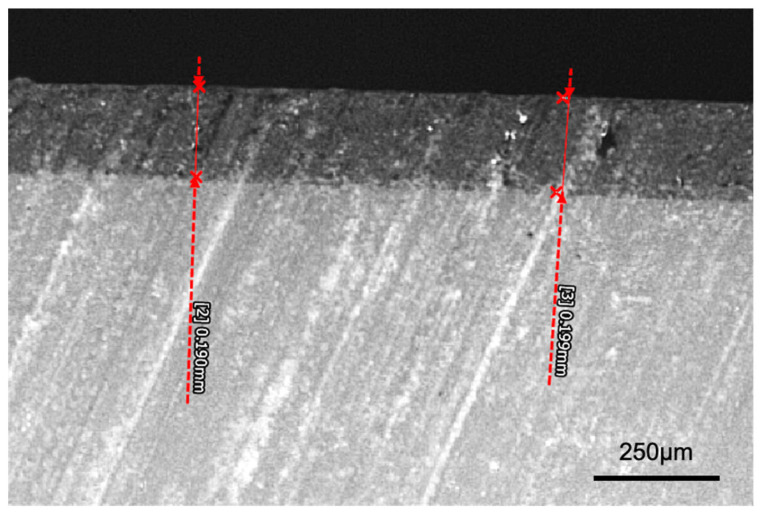
Representative (n = 1) cross-section of varnish layer (original magnification of 250×).

A cross-sectional light micrograph of the varnish layer is presented in Figure 7. This image demonstrates that the varnish layer was approximately 200 µm. The thickness of the varnish was slightly thinner at the edges of the specimens.

**Figure 8:**
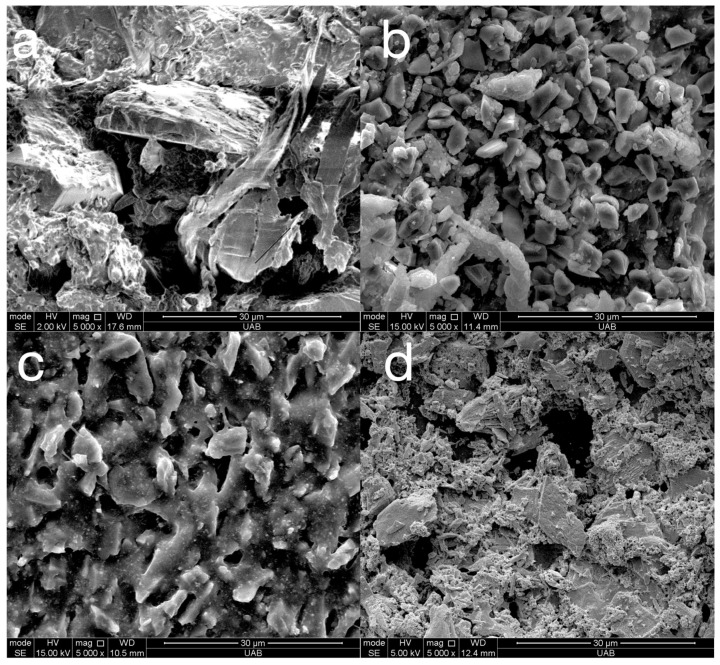
Representative (n = 1) SEM images (original magnification of 5000×) of different polishers: ASAP pre-polish (**a**), ASAP high-shine (**b**), rubber acrylic polisher (**c**), and diamond abrasive paste (**d**).

**Figure 9:**
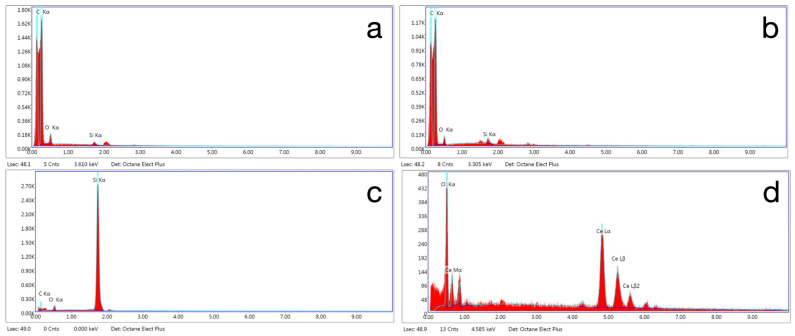
Representative (n = 1) EDS spectrum of abrasive particles from different polishers: ASAP pre-polish (**a**), ASAP high-shine (**b**), rubber acrylic polisher (**c**), and diamond abrasive paste (**d**).

Representative SEM images of the abrasive particles in the polishers are presented in Figure 8 and the elemental composition of the particles was identified by EDS as presented in Figure 9. The particles in both the pre-polish and high-shine polisher of the ASAP system are composed of carbon, indicating that they are diamond. The particles in the pre-polish step are around 30 µm and, in the high-shine step, are around 4 µm. There is also silicon and oxygen that may be part of silicone binder. The particles in the rubber acrylic polisher may be silicon carbide that are approximately 6 µm. The abrasive particles in the polishing paste contain cerium and oxygen, suggesting they are cerium oxide, a common abrasive for glasses, embedded in a binder. The particle size was approximately 10 µm.

## 4. Discussion

The results of this study demonstrate significant differences in final surface roughness and gloss based on both the surface treatment and the 3D-printed crown material used. Moreover, the effectiveness of each surface treatment varied depending on the material, leading to the rejection of the null hypothesis. Among the two polishing systems tested, the diamond-impregnated rubber polishers (ASAP) produced lower surface roughness than the bristle brush and abrasive paste system (MOD) for both OnX Tough 2 and VarseoSmile TriniQ. However, for VarseoSmile TriniQ, this difference in roughness was not statistically significant. In terms of gloss, the ASAP system achieved higher gloss on Rodin Sculpture, whereas the MOD system performed better on VarseoSmile TriniQ. The ASAP system was composed of diamond abrasives, which were more capable of polishing the harder surface of Rodin Sculpture than the softer silicon carbide and cerium oxide abrasives used in the MOD system. Previous studies have shown a negative correlation between surface roughness and gloss in restorative composites [13], a trend that held true for Rodin Sculpture but was less evident for VarseoSmile TriniQ.

Although the differences in roughness between polishers were statistically significant, they may not be clinically meaningful. Roughness values below 0.5 µm are generally imperceptible to the tongue [5], and values below 0.2 µm are considered sufficient to minimize biofilm accumulation [2]. In this study, all materials except some specimens of Rodin Sculpture polished with the MOD system after toothbrushing maintained roughness below 0.2 µm, suggesting limited clinical relevance for the observed differences. SEM images of the specimens reveal that they are relatively smooth before (Figure 4) and after (Figure 5) toothbrushing.

The Ra values achieved by the polishers in this study were lower than values obtained in a previous study of 3D-printed crown material, which achieved a polish of 0.371 µm with a goat hair brush and 0.781 µm with a silicone polisher. The experimental silicone polisher used in the previous study may not have been optimized for the 3D-printed material as it produced a higher roughness than without surface treatment (0.615 µm) [14]. Another previous study achieved similar Ra values as obtained in this study for a polished 3D-printed material (0.055 µm); however, they used 600-, 800- and 1200-grit sandpaper followed by a 0.1 µm diamond slurry [19].

Differences in gloss between polishing systems were more pronounced than differences in roughness. Gloss values of 60–70 GU are considered acceptable, 70–80 GU good, and values above 80 GU excellent [13]. Specifically for dental composites, gloss levels below 40 GU are regarded as clinically unacceptable, 50–80 GU as acceptable, and 90–100 GU as indicative of high gloss [1]. The MOD system achieved excellent gloss with VarseoSmile TriniQ (85 GU) but only borderline acceptable gloss with Rodin Sculpture (54 GU). The performance of the MOD system was significantly influenced by the material it was paired with, showing better results on softer, lower-filled crown materials. Hardness testing revealed that Rodin Sculpture exhibited nearly double the hardness (approximately 39 HV) compared to OnX Tough 2 and VarseoSmile TriniQ (both around 21 HV). Therefore, the hardness of a 3D-printed crown material may be an indicator for the best polishing system. For example, the MOD system may be appropriate for a material such as C&B MFH (NextDent, 3.1% filled) with a hardness of 14.1 HV but not ideal for a material such as Ceramic Crown (SprintRay, 49% filled) with a hardness of 42 HV [6]. A material with an intermediate hardness, such as OnX Tough (SprintRay, 37% filled) with a hardness of 29 HV, may or may not reach a high-gloss finish with the MOD system [6].

Observation of Figure 2 and Figure 3 reveals information regarding the efficacy of the different steps of the polishing systems. The first step of the ASAP system had a large effect on reducing roughness but did not affect gloss considerably. The second step of the ASAP system was responsible for producing a large improvement in gloss. This performance was expected, as the first step of the polishing system was labeled as a pre-polisher and contains large (30 µm) diamond abrasives, whereas the second step was labeled as the high-shine polisher and contains smaller (4 µm) diamond abrasives. For the MOD system, the first step (the bristle brush and polishing paste) was only effective in improving gloss and roughness for the less-filled materials, OnX Tough 2 and VarseoSmile TriniQ. The cerium oxide abrasive in the polishing paste may have not been as effective as the diamonds in the ASAP system with the highly filled Rodin Sculpture material.

The results of this study indicate only a slight increase in surface roughness across all materials after 20,000 cycles of toothbrushing. In contrast, a previous study reported an 85% increase in roughness after just 10,000 cycles [19]. Additionally, the current study observed a 22–40% (9.2–31.5 GU) reduction in gloss of polished specimens post-toothbrushing, whereas the previous study reported a 90% decrease [19]. A decrease in gloss of 9.2–31.5 GU in this study would be noticeable but not clinically unacceptable, as it exceeds the perceptibility threshold of 6.4 GU—the point at which 50% of observers can detect a difference—yet remains below the acceptability threshold of 35.7 GU, above which differences are generally considered unacceptable [20]. Both studies used the same sliding distance (10 mm) and frequency (2 Hz), although the applied load differed slightly—250 g in the previous study versus 350 g in the current one. The toothpaste slurry used in both studies maintained the same 2:1 water-to-toothpaste ratio, though the toothpaste in the current study had a higher relative dentin abrasivity (RDA) of 150 compared to 70 previously. The most notable procedural difference that may account for the greater increase in roughness and loss of gloss in the previous study is the brushing mechanism. The earlier study employed a device that provided both horizontal brushing and specimen rotation, while the current study used a device limited to horizontal brushing only. Observation of Figure 5 and Figure 6 show that there are only shallow dispersed scratches that occur after toothbrushing. The shallow, widely spaced scratches from toothbrushing can significantly reduce the gloss by scattering light and disrupting specular reflection, while having minimal impact on surface roughness (Ra) because they do not substantially alter the average surface height profile.

In this study, a filler-free varnish was applied to the surface of the specimens. The clinical advantage of using a varnish is that it can be applied more quickly and easily than the multi-step process required to polish a crown. The thickness of the varnish layer was found to be approximately 180 microns (Figure 7). A previous study reported the thickness of a different varnish (Optiglaze, GC) to be around 40 µm. As a result of its thickness, the application of a varnish may necessitate minor occlusal adjustments at areas of occlusal contact. A previous study evaluated the surface roughness achieved by two different varnishes (Optiglaze, GC and Akzent LC, Vita) on a 3D-printed crown material. It found that these varnishes produced a surface roughness comparable to that achieved with a goat hair polisher and lower than that obtained with silicone polishers [14]. These findings align with the results of the current study, where the varnish produced surface roughness equal to or lower than that obtained through polishing. Additionally, another study reported that 20,000 cycles of simulated toothbrushing did not significantly affect the surface roughness of 3D-printed crown materials coated with a varnish (Optiglaze, GC) [17]. This observation is consistent with the current study, where varnished specimens demonstrated superior retention of gloss and resistance to surface roughness compared to polished specimens after brushing.

This study has several limitations. First, only the Ra parameter was evaluated. A more comprehensive understanding of surface topography could have been achieved through three-dimensional surface roughness analysis, such as Sa (arithmetical mean height of a surface). Second, the crown materials in this study were not polymerized in vacuum, nitrogen, or glycerin. It is possible that any of these curing conditions may have affected the surface properties of the materials. Third, different printers were used for different materials. Differences in light intensity, wavelength, or exposure time between the printers may have altered the degree of polymer cross-linking within the printed materials, which, in turn, could influence their surface hardness and response to polishing. Fourth, the polishing sequence used for the MOD polishing system was based on consultation with its creator; however, not all steps of the system were used. Possibly, alterations in these polishing steps could lead to a high gloss on Rodin Sculpture. Fifth, one operator polished all specimens by hand and automated polishing or multiple operators may have improved generalizability of results. Future studies may evaluate additional materials and polishing protocols. Aside from roughness and gloss, the staining resulting from different polishing procedures should also be measured.

## 5. Conclusions

Several clinically relevant results were ascertained from this study, including:

1. The two-step diamond-impregnated rubber polishing kit (A.S.A.P. Indirect+ All Surface Access Polishers) could produce an equivalent gloss on all 3D-printed composite materials tested, whereas the polishing kit (MOD 3D-printed and milled acrylic polishing kit) including a bristle brush and abrasive paste achieved a better gloss with softer 3D-printed materials.
2. All polishing and varnishing techniques could achieve a clinically acceptable roughness (Ra < 0.2 µm) that was maintained even following 20,000 cycles of toothbrushing.
3. Application of a varnish (Rodin Glaze N2-Free) achieved a similar or better roughness and gloss than polishing. Following toothbrushing, varnished specimens maintained similar or better roughness and gloss than polished specimens.

## Figures and Tables

**Table 1:** 3D-printed crown materials used in this study and their properties (mean ± standard deviation).

Material	Manufacturer	Microhardness (HV)	Filler wt%
Rodin Sculpture	Pac-Dent	39.58 ± 1.90	59.1 ± 0.9
OnX Tough 2	SprintRay	21.28 ± 1.62	33.2 ± 1.6
VarseoSmile TriniQ	BEGO	21.13 ± 2.29	20.8 ± 0.7

**Table 2:** Properties of 3D-printed crown materials following different surface treatments (mean ± standard deviation).

		Roughness (µm)	Roughness After Brushing (µm)	Gloss (Gloss Units, GU)	Gloss After Brushing (Gloss Units, GU)
Rodin Sculpture	Varnish	0.04 ± 0.02 c	0.05 ± 0.03 c	70.5 ± 5.1 b	59.8 ± 3.9 b
	ASAP	0.08 ± 0.02 b, A	0.11 ± 0.01 b, A	77.6 ± 3.1 c	55.4 ± 5.5 b
	MOD	0.14 ± 0.02 a, A	0.19 ± 0.02 a, A	54.0 ± 2.5 a, A	28.6 ± 9.6 a, A
OnX Tough 2	Varnish	0.05 ± 0.02 b	0.06 ± 0.03	74.8 ± 7.9	63.8 ± 2.9 b
	ASAP	0.06 ± 0.01 b, AB	0.07 ± 0.01 AB	75.7 ± 5.3	55.3 ± 8.8 b
	MOD	0.08 ± 0.02 a, B	0.09 ± 0.02 B	71.3 ± 12.0 B	41.5 ± 5.5 a, B
Varseo-smile TriniQ	Varnish	0.04 ± 0.02 b	0.05 ± 0.03	70.5 ± 5.1 a	61.3 ± 4.0
	ASAP	0.05 ± 0.01 ab, B	0.06 ± 0.01 B	74.0 ± 4.0 a	57.8 ± 6.8
	MOD	0.07 ± 0.02 a, B	0.09 ± 0.01 B	84.6 ± 4.4 b, C	53.1 ± 9.5 C

Different lowercase letters in each column represent statistically different surface treatments within each material group. Different uppercase letters in each column represent statistically different materials within each surface treatment group.

**Table 3:** ANOVA table for roughness.

	Mean Square	df	F	*p*
Material	7 × 10^−3^	2	21.16	<0.001
Surface treatment	29 × 10^−3^	2	93.54	<0.001
Material × Surface treatment	5 × 10^−3^	4	16.21	<0.001

**Table 4:** ANOVA table for roughness after brushing.

	Mean Square	df	F	*p*
Material	19 × 10^−3^	2	40.13	<0.001
Surface treatment	38 × 10^−3^	2	80.65	<0.001
Material × Surface treatment	10 × 10^−3^	4	22.23	<0.001

**Table 5:** ANOVA table for gloss.

	Mean Square	df	F	*p*
Material	654.32	2	17.42	<0.0001
Surface treatment	258.91	2	6.90	0.002
Material × Surface treatment	900.52	4	23.98	<0.001

**Table 6:** ANOVA table for gloss after brushing.

	Mean Square	df	F	*p*
Material	682.21	2	17.11	<0.001
Surface treatment	3395.59	2	85.16	<0.001
Material × Surface treatment	444.35	4	11.14	<0.001

## Data Availability

The raw data supporting the conclusions of this article will be made available by the authors on request.
